# A homozygous splicing mutation in *ELAC2* suggests phenotypic variability including intellectual disability with minimal cardiac involvement

**DOI:** 10.1186/s13023-016-0526-8

**Published:** 2016-10-21

**Authors:** Nadia A. Akawi, Salma Ben-Salem, Jozef Hertecant, Anne John, Thachillath Pramathan, Praseetha Kizhakkedath, Bassam R. Ali, Lihadh Al-Gazali

**Affiliations:** 1 Department of Pathology, College of Medicine and Health Sciences, United Arab Emirates University, Al-Ain, United Arab Emirates; 2 Department of Paediatrics, Tawam Hospital, Al-Ain, United Arab Emirates; 3 Department of Paediatrics, College of Medicine and Health Sciences, United Arab Emirates University, P.O. Box 17666, Al-Ain, United Arab Emirates; 4 Present address: Wellcome Trust Sanger Institute, Wellcome Trust Genome Campus, Hinxton, UK

**Keywords:** *ELAC2*, Mitochondrial disorder, 5′ end unprocessed mt-RNAs, Splice site mutation, Intellectual disability, Respiratory chain complex I (RCCI) deficiency

## Abstract

**Background:**

The group of *ELAC2*-related encephalomyopathies is a recent addition to the rapidly growing heterogeneous mitochondrial disorders.

**Results:**

We describe a highly inbred consanguineous Pakistani family with multiple affected children in 2 branches exhibiting moderately severe global developmental delay. Using homozygosity mapping, we mapped the phenotype in this family to a single locus on chromosome 17. In addition, whole-exome sequencing identified a homozygous splicing mutation (c.1423 + 2 T > A) in *ELAC2* gene that disrupted the canonical donor splice site of intron 15 of all known isoforms. A noticeable reduction in ELAC2 expression was observed in patients compared to controls. In addition, patients exhibited significantly increased levels of 5′ end unprocessed mt-RNAs compared to the control fibroblast cells.

**Conclusions:**

The only three previously reported families with defects in *ELAC2* gene exhibited infantile hypertrophic cardiomyopathy and complex I deficiency. In contrast, our patients exhibited intellectual disability as the main feature with minimal cardiac involvement. Therefore our findings expand the phenotypic spectrum of *ELAC2*- associated disorders illustrating clinical heterogeneity of mutations in this gene. In addition, ELAC2 mutations should be considered when evaluating patient with mainly intellectual disability phenotypes.

**Electronic supplementary material:**

The online version of this article (doi:10.1186/s13023-016-0526-8) contains supplementary material, which is available to authorized users.

## Background

Mitochondria are the key suppliers of cellular-energy through five protein complexes known as respiratory chain complexes (RCCI, RCCII, RCCIII, RCCIV, RCCV). These complexes catalyze the oxidation of nutrients and the associated energy transduction into ATP *via* a pathway known as oxidative phosphorylation. Mitochondrial disorders refer to a group of extremely heterogeneous multisystemic clinical presentations. These disorders are also known as mitochondrial encephalomyopathies since they are almost always involving the muscle and the brain, where energy is highly required [1]. Other clinical manifestations such as blindness, deafness, and movement disabilities may also be present in the spectrum of the mitochondrial group of disorders. Mitochondrial encephalomyopathies are mainly caused by mutations that directly affect the maternally inherited mitochondrial DNA (mt-DNA). In addition, a considerable number of the mitochondrial encephalomyopathies were found to be associated with mutations in nuclear genes and hence segregating in autosomal recessive or dominant patterns [1, 2]. The pathogenic mutations in nuclear genes mostly encode enzymes implicated in the replication, transcription or translation of the mt-DNA and therefore, affecting the biogenesis and maintenance of the mitochondria [1, 2].

Recently, whole-exome sequencing has been used to unravel the genetic defect underlying a multisystemic mitochondorial disorder in three unrelated families [3]. The phenotype of these patients included infantile hypertrophic cardiomyopathy, developmental delay, lactic acidosis and RCCI deficiency (MIM 252010). The authors identified different compound heterozygous and homozygous pathogenic mutations in *ELAC2* gene (MIM 605367) as the underlying causes in these families. *ELAC2* is a nuclear-gene located on the short arm of chromosome 17 encoding Zinc phosphodiesterase ELAC protein 2 (tRNaseZ 2). The encoded protein has a mitochondrial tRNA 3′-processing endonuclease activity. It plays a key role in mitochondrial tRNA maturation by removing a 3′-trailer from precursor tRNA, a crucial step in tRNA processing [4].

In this paper, we report a large highly inbred Pakistani family of Baluchi origin with five individuals in two branches affected by intellectual disability and developmental delay. We mapped the disease-causing gene in this family to a segment on chromosome 17 and identified a homozygous splicing mutation in *ELAC2* gene. Although previous mutations in this gene were found to cause hypertrophic cardiomyopathy and complex I deficiency, affected individuals in this report had minimum cardiac involvement with intellectual disability and developmental delay being the main presenting features. Therefore, the clinical and molecular data described in this manuscript expand the phenotypes associated with *ELAC2* mutations and highlight the clinical heterogeneity of *ELAC2*-related mitochondrial disorders.

## Methods

### Research subjects

**Fig. 1:**
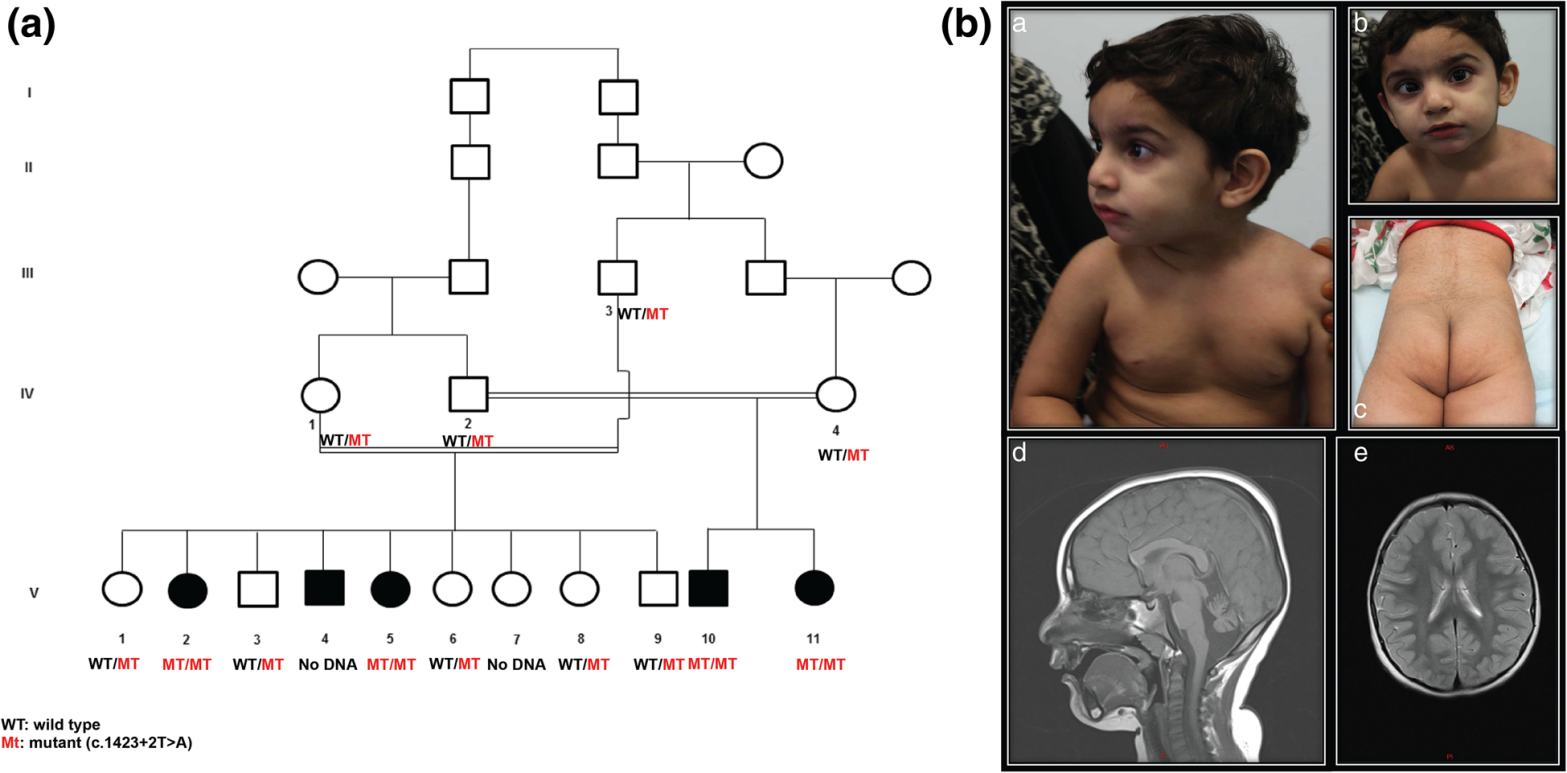
Pedigree of the family and key clinical features of patient V10 in this study. **a**) The main characteristics of autosomal recessive inheritance including consanguinity and multiple affected children of both sexes can be seen in this pedigree. *Circles and squares denote females and males respectively, filled symbols represent affected members, double lines denote consanguineous marriage. WT: wild-type; Mt: mutant for c.1423 + 2 T > A.* **b**) *a* & *b*, Front and lateral facial photographs of patient V10 showing squint, bulbous nose, low set ears with simple helix and mild micrognathia. *c*, Asymmetric buttocks of patient V10 with the right side smaller and the fold is higher than the left. *d*, T1 saggital view showing cerebellar hypoplasia. e, T2 axial view showing diffuse increased signal in white matter

Multiple affected children in two branches of a consanguineous family exhibiting severe developmental delay with very mild hypertrophy of the interventricular septum have been evaluated (Fig. 1). The study was approved by Al-Ain District Human Research Ethics Committees (protocol number 10/09) and the family provided a written informed consent for participating in the study.

### Genome-wide SNP genotyping and homozygosity mapping

Genomic DNA was isolated from peripheral blood collected in EDTA tubes from all the family members using the Flexigene DNA extraction kit (Qiagen, USA). Genotyping of their whole genome was undertaken using GeneChip Genome-Wide Human SNP Array 6.0 (Affymetrix, USA). SNP genotypes were obtained by following the standard protocols supplied by the manufacturer. Genotypes were called with the Genotype Console program (Affymetrix, USA). Generated SNPs derived from the family members’ DNA were loaded into the software package HomozygosityMapper (http://www.homozygositymapper.org) and subjected to homozygosity mapping analysis [5].

### High-throughput sequencing of nuclear and mitochondrial DNA

Whole-exome sequencing of the extracted nuclear DNA was performed by Oxford Gene Technology (Oxfordshire, UK). Nuclear DNA was extracted from the blood of patients V2 and V10 using Flexigene DNA extraction kit (Qiagen, USA). Exome capturing and enrichment was carried out using SureSelect All Exon V4 kit (Agilent Technologies, USA) following the manufacturers’ protocols. Whole-exome sequencing was carried out on Illumina HiSeq 2000 system (Illumina). All the variants were mapped, annotated and filtered as described previously [5]. Sequencing of the mt-DNA was carried out as a service by Nijmegen Centre for Mitochondrial Disorders (NCMD, the Netherlands). The complete mt-DNA was isolated from DNA extracted from the skin fibroblasts of patient V10. The mt-DNA (Genbank accession# NC_012920.1, 16569 bp) was screened for rearrangements and mismatches using the Ion torrent personal genome machine (Life Technologies, USA).

### Transcript analysis

Total RNA was isolated from fresh blood with QIAamp RNA blood kit (Qiagen, USA). Single-stranded cDNA was synthesized from the same starting materials of RNA (1 μg) using the GoScript reverse transcription system in accordance with the manufacturer’s instructions (Promega, USA). To avoid genomic amplification, reverse transcription nested-PCR was carried out with primers spanning the exon–exon junctions of NM_018127.6. The primers for the first round PCR F: 5′ TGTGAGAATGCCACCTTTCA 3′ and R: 3′ GCACCAGACAGGTCTGAAACT 5′ generating product of 967 bp in size. The primer for the second round nested-PCR NF: 5′ CACCAGTTTCCGCTGTAAGA 3′ and NR: 3′ CAAGGCGCGTTCTCTCTG 5′ generating product of 499 bp in size. The nested-PCR products were separated on 2 % agarose gels.

### Sanger DNA sequencing

Direct DNA sequencing was carried out using the BigDye Terminator kit v3.1 (Applied Biosystems, USA). PCR amplification products were sequenced using the DNA sequencing with fluorescent automated sequencing on the ABI 3130*xl* genetic analyzer (Applied Biosystems, USA). Sequencing data were analyzed using ClustalW2 referencing the NM_018127.6 for *ELAC2* gene Refseq sequence.

### Fibroblast culturing

Skin biopsy from patient (V10) and normal control were sliced into smaller pieces and cultured in 6 well plates as described previously [6].

### Quantitative PCR (QPCR) of mt-RNAs of *ND1, ND2* and *ATP8* genes

Total RNA was extracted from 1 × 10^6^ cells using Qiazol reagent (Qiagen, USA) following the manufacturer’s instructions. The expression levels of 5′ end unprocessed mt-RNAs for *mtATP8, mtND2,* and *mtND4* genes were inspected in fibroblasts from case 1 (V10), and four healthy control samples by means of qPCR using the QuantStudio® 7 Flex Real-Time PCR System (Applied Biosystems). Primers used in the qPCR for mt-RNAs and *HPRT1* genes were designed as described by Haack et al. [3]. Primers bind to the 5′ region for tRNA^Lys^ (junction ATP8/6), for tRNA^Arg^ (Junction ND4L/ND4), and for tRNA^Leu(UUR)^ND1. The human *HRPT* was used as an internal control, and all experiments were run in quadruplicates and repeated twice to ensure reproducibility. All reactions were amplified and quantified in a total volume of 20 μl. The reactions contained 2X SYBR Green PCR master mix (cat#4309155, Applied Biosystems, USA), 200nM of each primer, and 1 μl of the cDNA samples. Standard PCR condition was used as follows: 10 min activation at 95 °C, followed by 40 cycles of amplification at 95 °C for 15 s and 60 °C for 1 min. Data analysis including the threshold cycle (CT) and relative quantification (RQ) values were calculated using the QuantStudio® 7 Flex analysis SDS software (Applied Biosystems, USA).

### Western blots

Total protein was extracted from the skin fibroblasts of patient V10 and two different healthy controls using radioimmunoprecipitation assay (RIPA) buffer containing protease and phosphatase inhibitors (Thermo Scientific, USA). Protein was also extracted from HEK293T cells lysate as an additional control. Protein concentration was determined by the bicinchoninic acid assay (BSA; Sigma, USA), and ~50ug protein lysates were separated on a 8 % SDS PAGE and transferred to nitrocellulose membrane. The blots were blocked in 5 % milk in Phosphate Buffered Saline with Tween 20 (PBST) and probed with rabbit anti-ELAC2 antibody (1:100; sc-138774, Santa Cruz, USA) overnight. Secondary antibody (anti-rabbit; Santa Cruz, USA) was used at a dilution of (1:5000). The blots were developed with ECL plus reagent and imaged in Typhoon FLA 7000 (GE Healthcare Life Sciences, Canada). The blots were stripped and re-probed with a mouse monoclonal antibody against Tubulin (1:10,000; T5168, Sigma, USA) which served as a loading control. Densitometric analysis of protein bands was performed using Image Studio Lite software (LI-COR) and graph was generated using GraphPad Prism software.

## Results

### The five affected children in the studied family exhibited global developmental delay

The affected family is highly inbred Pakistani family of Baluchi origin (Fig. 1A). The parents of the index case (V10) are second cousin with two affected children. In another branch of the family there were three affected individuals, two girls and a boy.

Case 1 (V10) was a 4 years old boy, the product of normal pregnancy and delivery. His birth weight was 3 kg (25^th^ centile). No other measurements were available. There were no neonatal problems. At the age of 3 months he had focal left lower limb myoclonic seizure at night which lasted 2–3 min. He was noted by the parents to have delayed development at the age of 5 months. He had no head control and he was unable to roll over. At the age of 9 months he had seizure-like episodes 3 times with each of them lasting 1 min. These attacks disappeared and did not recur. However, he had attacks of head nodding to the left side several times per day lasting 5 min each. He was evaluated by us at the age of 3 years because of delayed development. At this age he was able to walk holding furniture and was saying 3–4 words only. On examination his weight was 11.170 kg (<5^th^ centile), height 82.90 cm (<5^th^ centile), head circumference 45.50 cm (−4.5 SD). He had left estropia, subtle dysmorphic features including bulbous nose, thin upper lip, mild micrognathia and low set ears with simple helix. Deep tendon reflexes were exaggerated (Fig. 1B-a&b). The fat distribution was not symmetrical at the buttock area (Fig. 1B-c). The nipples were not inverted. The rest of the examination was normal. The magnetic resonance imaging (MRI) of the patient’s brain showed reduced volume of the cerebellar vermis and hemispheres with widening of posterior fossa and extra-axial cerebrospinal fluid spaces (Fig. 1B-d&e). Diffused abnormal areas of low density bilaterally at the internal capsule and frontoparietal white matter were also seen (Fig. 1B-e). Echocardiography showed borderline hypertrophy of the interventricular septum. The brain electroencephalogram (EEG) was reported to be normal. Serum ammonia, amino acid chromatography and urine organic acids were all normal. Lactic acid was slightly elevated. Transferrin isoelectric focusing was normal. Comparative genomic hybridization (CGH) array revealed an interstitial duplication of 79 oligonucleotide probes from 7q11.3-q21.11 spanning approximately 1.7 MB. The duplicated interval contains approximately 16 known genes. However, no information regarding a phenotype associated with duplication of any of these genes is currently available. Fluorescence *in situ* hybridization (FISH) testing of a maternal sample demonstrated that this duplication was inherited from the mother and was absent in the other affected siblings indicating that it is a familial variant without phenotypic significance. Mitochondrial enzymes (complexes I, II, III, IV, V, pyruvate dehydrogenase, citrate synthase) measurements in the patient’s (V10) blood and fibroblast were found to be normal at the age of 5 years.

Case 2 (V11), was the sister of case 1 (V10) . She was the product of normal pregnancy and delivery. Birth weight was 2.8 kg (3^rd^ centile). There were no neonatal problems. She was noted to have developmental delay in the first few months of life. Evaluation at the age of 2.5 years revealed a weight of 12.94 kg (10 centile), height 84.2 cm (5^th^ centile), and head circumference of 42.4 cm (−6.5 SD). She had similar subtle dysmorphic features as her brother. She was unable to walk and had no speech. Detailed eye examination was normal. Echocardiography revealed some degree of hypertrophy of the interventricular septum. Array CGH was normal. Lactic acid was normal.

There were three other affected individuals in another branch of the same family (Fig. 1A). They were aged 19 years female (V2), 17 years male (V4) and 15 years female (V5). All had microcephaly and were unable to walk but can crawl and had 3–4 words sentences. Unfortunately, they lived in a remote region and we were, therefore, unable to evaluate them further.

### Genome-wide genotyping of the two branches in the studied family mapped the disease to a single locus on chromosome 17

**Fig. 2:**
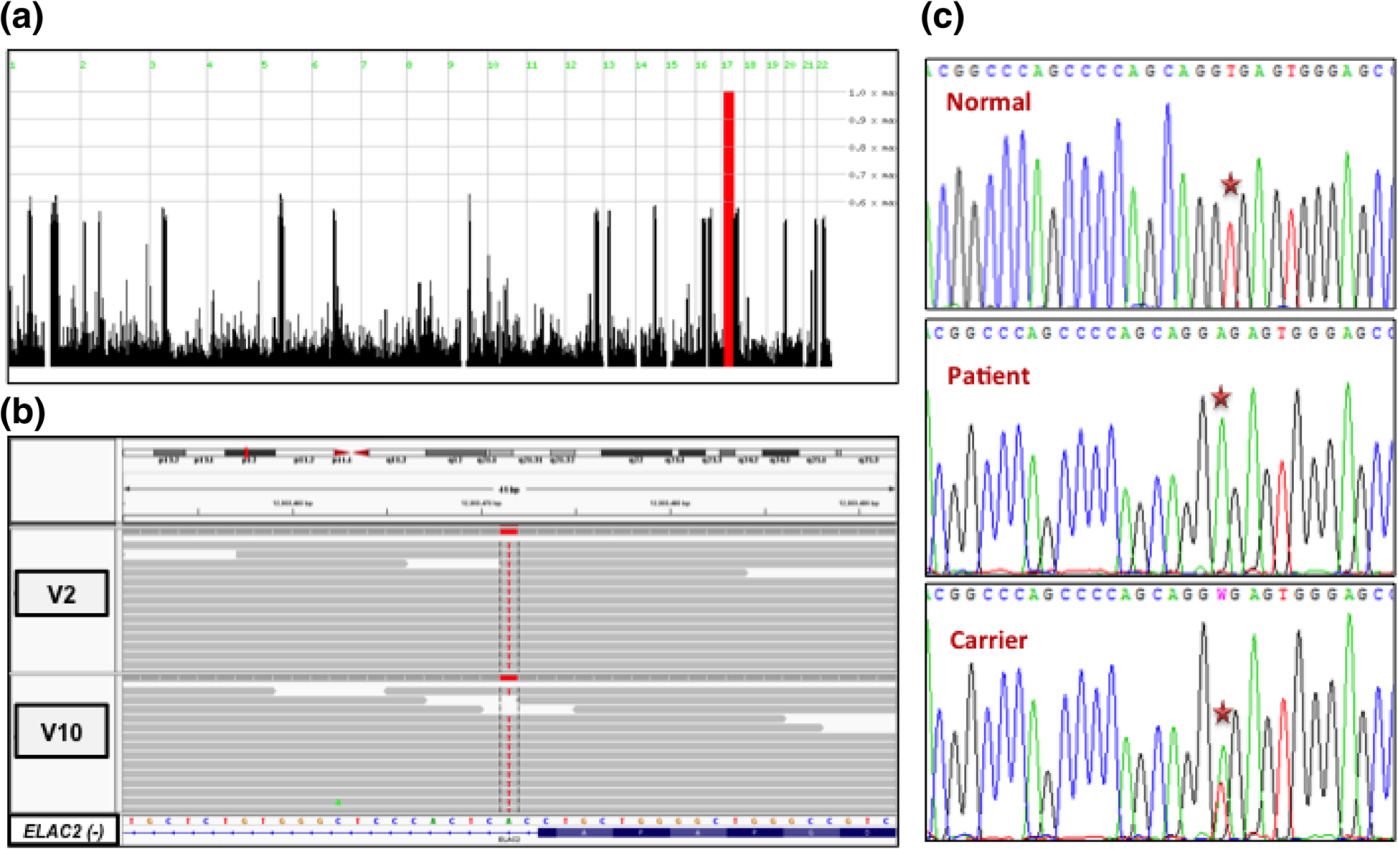
Genome-wide genotyping and sequencing results. **a**) Genome-wide homozygosity mapping analysis revealed one stretch of homozygous genotypes in all the investigated patients on chromosome17 (indicated by a red bar). **b**) Whole-exome sequencing IGV of the affected children V2 and V10 showing a 1 bp substitution of a canonical splice site in all reads of *ELAC2* exon 15 . *Sequence of wild type gene on reverse strand (-) and exon annotation at the bottom.* **c**) Sanger sequencing verified that the c.1423 + 2 T > A mutation is homozygous in patients (Patient), heterozygous in parents and some of the unaffected siblings (Carriers) and absent in 100 normal controls (Normal)

To identify the disease locus, whole genome SNP genotyping was performed for two affected children (V2 and V10), two parents (IV1 and IV2) and five unaffected siblings (V1, V3, V6, V8 and V9). Homozygosity mapping analysis of the generated genotypes revealed a single homozygous region on chromosome17 (11,496,228–43,871,147) flanked by rs17514650 and rs12944712 (Fig. 2a). This 32.4 Mb genetic interval encompassed 796 genes. Using additional genotyping data from family members, we narrowed down the interval to 4.37 Mb flanked by rs17514650 and rs17715109.

### Next-generation sequencing revealed a splicing aberration in the nuclear gene *ELAC2* while their mtDNA was normal

In order to reveal the molecular basis of the phenotype in the studied family, whole-exome sequencing was carried out on two affected children V2 and V10. A minimum of 72.53 % of the on-target regions was covered to a depth of at least 20×. Around 88,000 variants from the reference genome were identified. Of which 11,000 variations were of serious consequences on the corresponding protein products. After filtering out all the heterozygous reported variations, around 229 variations were left. Within the mapped disease-locus, a splicing mutation (chr17:12,903,471A > T) was found to be unique and shared between the two affected children (Fig. 2b). The splicing mutation changed a canonical donor splice site at the 5′ end of intron 15 of *ELAC2* gene. Substituting GT by GA at the splice site between exon 15 and intron 15 of *ELAC2* cDNA (NM_018127.6:c.1423 + 2 T > A) most probably disrupt its splicing. This splicing site was found to be shared between all the known UCSC and RefSeq isoforms of *ELAC2* gene and highly conserved in mammals (Additional file 1: Figure S1). The aberration was predicted to be disease-causing by MutationTaster prediction program. This mutation was not reported in any of the human nuclear genome databases such as dbSNP, the 1000 Genomes, the NHLBI exome variant database (http://evs.gs.washington.edu/EVS/), and ExAC browser (http://exac.broadinstitute.org/). In addition, it was neither found in the in-house exomes of individuals exhibiting intellectual disabilities, nor in the GalaxC database (allele frequency database of Arab disease mutations; https://galaxc.sengenics.com/galaxc/). The segregation of this mutation was verified using Sanger DNA sequencing and found to segregate well with the disease in the two branches of the family of this study (Fig. 2c). Both couples and all the unaffected individuals were heterozygous for this mutation. The novelty of the variant was also confirmed by its absence in 200 ethnically matched normal control chromosomes using Sanger sequencing.

To inspect any possible defects in the mitochondrial genome, the whole mtDNA of patient V10 was sequenced revealing three rare sequence variations that do not belong to the patient’s haplogroup. The m.09067A > G (p.Met181Val in MT-ATP6) sequence variation is mentioned in the human mitochondrial genome databases MITOMAP’s (0.08 %) and mtDB (0.07 %). This variant is predicted to be benign by mutation prediction programs including SIFT and PolyPhen. The m.16207A > G (D-Loop) sequence variation is mentioned in MITOMAP’s (0.27 %) and mtDB (0.21 %). The m.16318A > C (DLoop) sequence variation is mentioned in MITOMAP’s (0.04 %) and mtDB (0.11 %). Both variants are located in the non-coding region of the mtDNA.

### The c.1423+2T>A mutation in *ELAC2* altered its mRNA splicing pattern and reduced its protein expression

**Fig. 3:**
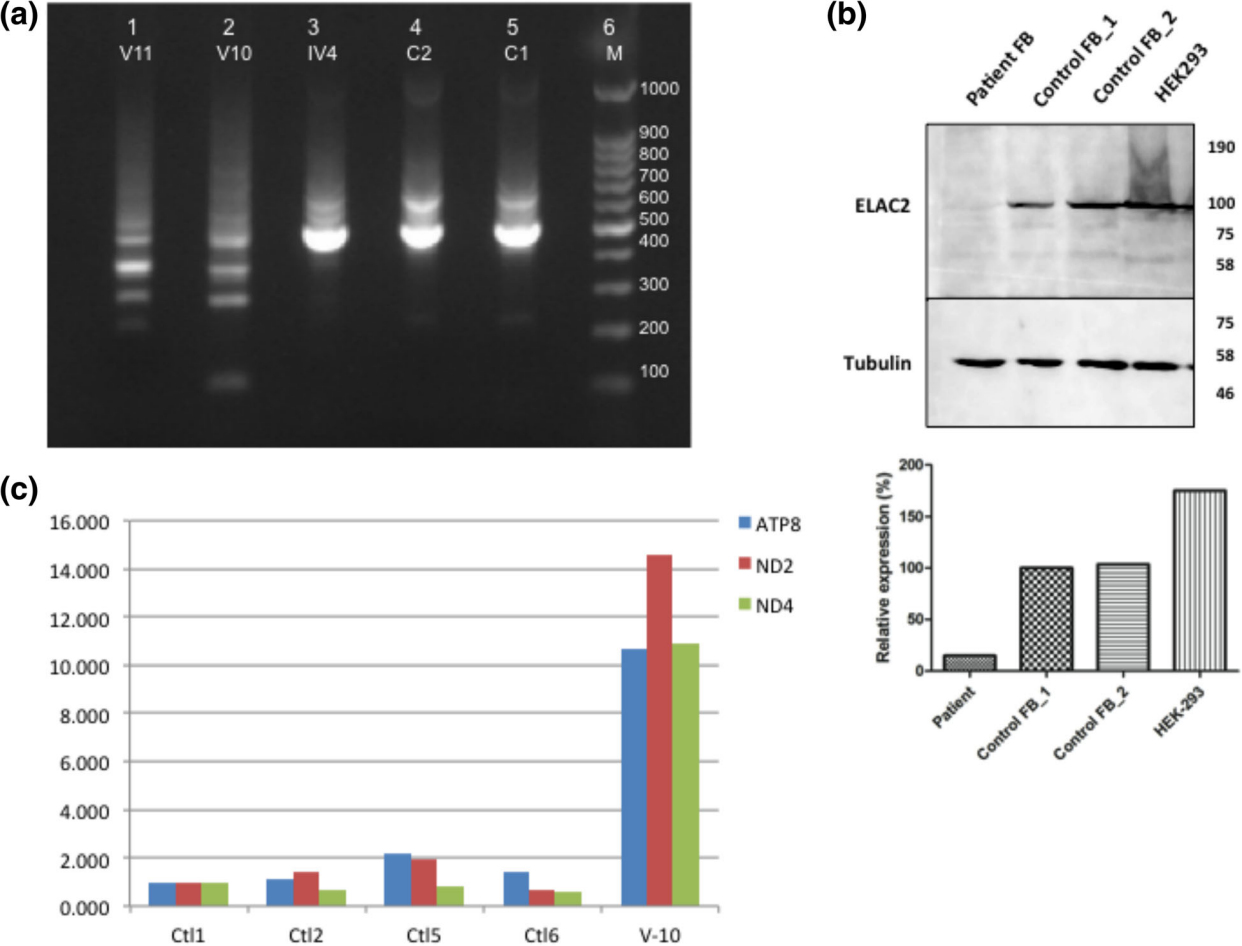
Effect of the splicing mutation on ELAC2 expression and on selected mitochondrial genes. **a**) The amplification products of *ELAC2* cDNA from patients, controls and parents were seen on a 2 % agarose gel. Bright bands were detected in the lanes of two healthy controls (C1 and C2) and the mother (IV4) at 500 bp (according to the DNA size marker M). Multiple fainter bands were seen in the patients’ (V10 and V11) lanes suggesting diminished expression of the normal WT transcript and the presence of other abnormal splicing products. **b**) Expression analysis of ELAC2 protein in patient fibroblasts. Total protein lysates from patient (V10) and two different control fibroblasts were analyzed for ELAC2 protein expression by immunoblotting against an antibody specific for ELAC2 isoform1. HEK293T cell lysate was used as a positive control. Mouse Tubulin antibody was used as a loading control. The levels of the protein was negligible in patient fibroblast compared to the control. Densitometric analysis of ELAC2 protein bands normalized to Tubulin levels, revealed that ELAC2 protein expression in patient fibroblasts was 14 % of that detected in control fibroblasts. **c**) A significant difference is seen between relative expressions of different unprocessed mitochondrial transcripts *mtATP8, mtND2,* and *mtND4*, in skin fibroblasts from patient V10, compared to four different control samples (Ctl1, Ctl2, Ctl5, and Ctl6). *The mRNA expression values were normalized to an internal control HPRT. X axis depicts quantitative expression; Y axis represents bar chart for controls, and patient samples, respectively*

To investigate the consequences of the detected splicing defect, total RNA was isolated from the leukocytes of two normal controls (C1 and C2), the mother (VI4) and the patients (V10 and V11) (Fig. 3a). Amplification of *ELAC2* cDNA in all the investigated individuals showed bright bands at around 499 bp in controls and the mother, whereas multiple fainter bands were seen in the patient’s samples (Fig. 3a, lanes 1 & 2). This result suggested a marked reduction in the abundance of the *ELAC2* normal transcript in patients.

Western immune-blotting was used to asses the effect of the splicing mutation on the expression of the protein product in the patient’s skin fibroblasts. ELAC2 band of around 90 KDa was detected in all the investigated control lysates but not in the patient’s fibroblast (Fig. 3b). The normalized ELAC2 expression level in patient’s fibroblast was found to be 14 % of that of the controls’.

### The 5′ end unprocessed mt-RNAs in patient’s fibroblasts are significantly elevated

Quantitative expression of mitochondrial transcripts corresponding to unprocessed *ATP8, ND2,* and *ND4* genes from case 1 (V10), and four healthy controls were performed using the QuantStudio® 7 Flex Real-Time PCR. Results showed significant increase in the levels of expression of unprocessed mitochondrial transcripts (tRNA^Lys^/*ATP8, tRNA* ^*Arg*^ */ND2*, and *ND4/tRNA* ^*Leu*(UUR)^) in the patient’s sample (V10) normalized to an internal control *HPRT* and compared to healthy control samples (Ctl1, Ctl2, Ctl5, and Ctl6) (Fig. 3c). For *tRNA* ^*Arg*^ */ND2,* the expression levels is approximately 13 times higher than control samples. Moreover, unprocessed tRNA^Lys^/*ATP8* and *ND4/tRNA* ^*Leu*(UUR)^ levels is almost 10 times higher than controls.

## Discussion

The human circular double–stranded mitochondorial genome is transcribed as large polycistronic transcripts from both strands [1, 7]. These transcripts are then processed to generate separate 13 messenger (m), 2 ribosomal (r) and 22 transfer (t) RNAs. The mRNAs encode for the RCC subunits and the rRNAs and tRNAs are required for their translation. Processing of the precursor transcripts is achieved via the cleavage of the 5′ and 3′ termini of each intervening tRNAs, leading to the separation of the adjacent mRNAs and rRNAs. The 3′ end processing of the tRNAs is mainly carried out by the mitochondorial endoneoclease tRNaseZ 2 encoded by *ELAC2* gene [4, 8]. Mutations in this gene were found to be associated with infantile hypertrophic cardiomyopathy, lactic acidosis and isolated RCCI deficiency in skeletal muscle in multiple unrelated patients [3]. The authors identified compound heterozygous missense (p.T520I) and nonsense (p.R211*) mutations in two siblings of a non-consanguineous family (Table 1). In addition, two homozygous missense mutations (p.F154L and p.L423F) were detected in three patients from two unrelated consanguineous families (Table 1). Patients’ tissue samples showed accumulation of unprocessed mt-tRNA intermediates that could be rescued by expression of wild type *ELAC2*. The findings were consistent with impaired 3-prime end processing of mt-tRNAs [3]. Although levels of mature mt-tRNA, mt-mRNA, and mt-rRNA were normal, patient cells showed increased levels of unprocessed mt-mRNA and mt-rRNA precursors and evidence of decreased translation of mitochondrial proteins. Haack et al. concluded that impaired RNase Z activity of ELAC2 causes a fatal failure in cellular energy metabolism by interfering with normal mitochondrial translation [3].

**Table 1:** Summary of clinical features of patients with *ELAC2* mutations

Families	F1		F2	F3		F316				
Ethnic	German		Arabic	Turkish		Pakistani				
Patients	#61525 (II-2)	#57415 (II-3)	#61982 (II-5)	#36355 (II-1)	#36355 (II-2)	V-2	V-4	V-5	V-10^a^	V-11
Sex	M	M	F	F	F	F	M	F	M	F
Clinical features										
Microcephaly	+	+	?	?	?	?	?	?	+	+
Psychomotor retardation	+	+	?	?	?	+	+	+	+	+
Muscle hypotonia	-	+	+	-	+	?	?	?	+	+
HCM	+	+	+	+	+ later DCM	?	?	?	Mild septal Hypertrophy	Mild septal Hypertrophy
Brain MRI	NA	Hyper-intensities in basal ganglia	NA	NA	Normal at 20 months	NA	NA	NA	Reduced volume of cerebellar vermis + hemispheres	NA
Lactic acid level in blood	Increased	Increased	Increased	Increased	?	NA	NA	NA	Slightly increased	NA
Mitoch. function in muscle	NA	Decreased complex I	Decreased complex I	Decreased complex I, IV	Decreased complex I, IV	NA	NA	NA	NA	NA
Course	death at 6 months	Alive at 2.10 years	death at 11 months	alive at 13 years	death at 4.9 years	alive at 19 years	alive at 17 years	alive at 15 years	alive at 4 years	alive at 2.5 years
mtDNA	normal	NA	NA	NA	NA	NA	NA	NA	normal	NA
Zygosity	Compound Heterozygous		Homozygous	Homozygous		Homozygous				
DNA change	c.[631C > T; 1559C > T]		c.460 T > C	c.1267C > T		c.1423 + 2 T > A				
Protein change	p.[Arg211^a^; Thr520Ile]		p.Phe154Leu	p.Leu423Phe		frameshift				
References	Haack et al. 2013 [3]					This study				

*HCM* hypertrophic cardiomyopathy, *DCM* dilated cardiomyopathy, *MRI* magnetic resonance imaging, *Mitoch*. mitochondrial. *Abbreviations*: *M* male, *F* female, + present, - absent, *NA* not available, ^a^ index patient, ? no materiel available

The main clinical feature in all the reported patients was severe early-onset hypertrophic cardiomyopathy (<6 months), whereas developmental delay with varying degrees of severity was observed in four out of the five patients (Table 1). Abnormal MRI images, muscular hypotonia, reduced head control, and delays in motor development were also common features. Three out of five died early (6 months, 11 months, 4 years) from cardiac failure, while two were still alive (2 years, 13 years) showing constant developmental delay. Lactate levels in blood were elevated and complex I deficiency was detected in muscles of all the investigated children [3].

In this paper, we present a consanguineous family with several affected individuals exhibiting severe psychomotor developmental delay, muscular hypotonia associated with subtle dysmorphic facial features. Structural brain abnormalities were present in the studied patients (V10 & V11) who had brain imaging. Echocardiography on the two available affected children (V10 & V11) showed mild hypertrophy of the inter-ventricular septum. Mapping the disease in the two branches of this family revealed a single large homozygous block on chromosome 17 that is shared by patients but not by the unaffected family members. Sequencing all the coding-exons of the nuclear genes in two affected children from the different branches of the investigated family pointed out one potential pathogenic mutation (c.1423 + 2 T > A) in *ELAC2*. The mutation discovered in the nuclear gene *ELAC2* disrupted a canonical splice donor site and therefore was predicted to cause a severe damage to the nascent transcripts. The splicing pattern of *ELAC2* mRNA was clearly different and its protein expression was drastically reduced in patients compared to normal controls. Mitochondrial enzyme studies were normal. However, quantification of 5′ unprocessed mt-RNAs showed significantly increased levels in the patient fibroblast sample as compared to healthy controls. This finding confirmed the implication c.1423 + 2 T > A in this disorder. Unfortunately, parents did not agree to take muscle biopsy from patient. Moreover, the main feature in our patients is severe intellectual disability with asymptomatic minimum septal hypertrophy while the patients reported by Haack et al. presented with severe cardiac symptoms due to hypertrophic cardiomyopathy, which led to death in some of them [3].

## Conclusion

In conclusion, the absence of significant cardiomyopathy and the presence of moderately severe intellectual disability as the main feature in this family suggest that aberrations in *ELAC2* should be considered in children with intellectual disability as the sole presenting feature. Additional *ELAC2* mutations will be helpful for elucidating the complex relationships between genotypes and the diverse clinical phenotypes in terms of severity, age of onset, and spectrum of organ system involvement for *ELAC2*-related disorders.

## Additional file

Additional file 1: UCSC genome browser. (DOCX 432 kb)

## Abbreviations

ATP: Adenosine triphosphate
CGH: Comparative genomic hybridization
CT: Threshold cycle
EEG: Electroencephalogram
*ELAC2*: elaC ribonuclease Z 2
FISH: Fluorescence *in situ* hybridization
MRI: Magnetic resonance imaging
mRNA: Messenger RNA
mt-DNA: Mitochondrial DNA
RCCs: Respiratory chain complexes
RQ: Relative quantification
rRNA: Ribosomal RNA
SD: Standard deviation
tRNA: Transfer RNA
