# Supplementary material for: A homozygous splicing mutation in ELAC2 suggests phenotypic variability including intellectual disability with minimal cardiac involvement
Source: Orphanet J Rare Dis. 2016 Oct 21;11:139. doi: 10.1186/s13023-016-0526-8 (PMC5073853; doi:10.1186/s13023-016-0526-8)
Supplement: Additional file 1: — UCSC genome browser. (DOCX 432 kb) [file 13023_2016_526_MOESM1_ESM.docx]

Additional file 1: Figure S1


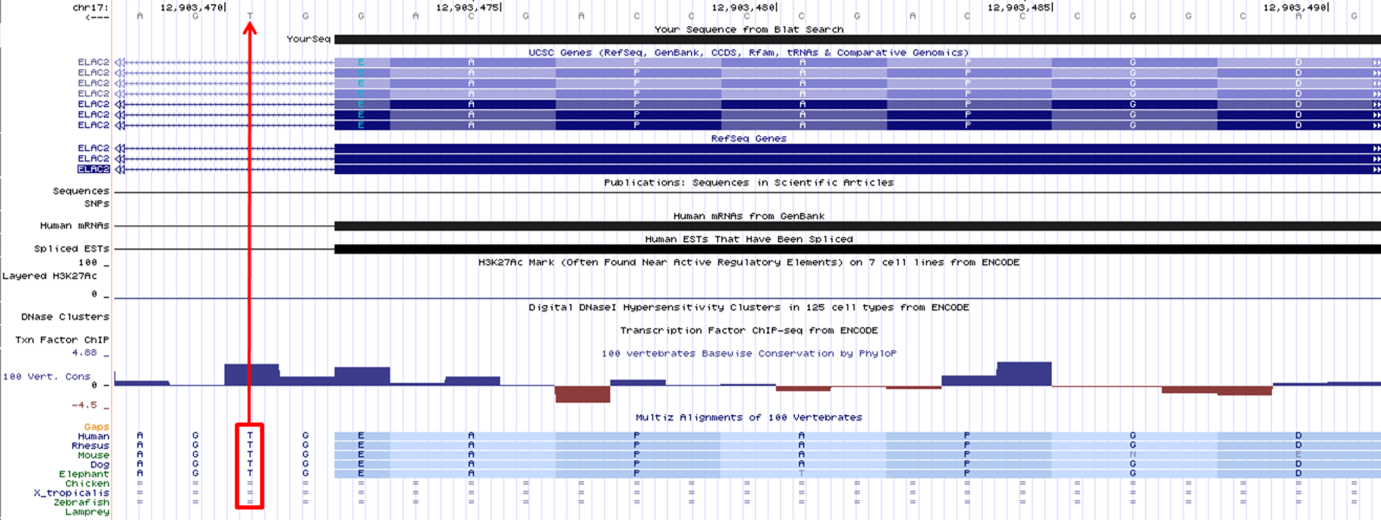


In the UCSC genome browser the substituted nucleotide (enclosed in a red square) was found in all the known isoforms of *ELAC2* and highly conserved in mammals.
